# RISKS AND HEALTH OUTCOMES OF NEW ONSET OF UNMET NEEDS FOR HELP WITH ACTIVITIES OF DAILY LIVING

**DOI:** 10.1093/geroni/igad104.2074

**Published:** 2023-12-21

**Authors:** Laura Sands, Maham Khan, Pang Du

**Affiliations:** Virginia Tech, Blacksburg, Virginia, United States; Virginia Tech, Blacksburg, Virginia, United States; Virginia Tech, Blacksburg, Virginia, United States

## Abstract

Twenty percent of community-living older adults who have difficulty completing mobility and self-care daily activities have unmet need for help with one or more daily activities. Prior research described correlates of existing unmet needs but did not consider which health characteristics and events increase risks for new onset of unmet needs. Data are from the 2011-2019 annual interviews of the National Health and Aging Trends Study. Onset of unmet need was calculated as having more unmet needs in the subsequent year compared to the prior year. Linear Mixed models were computed to assess which health characteristics and events increased risk for new onset of unmet need across 15,334 observations from persons who needed help with mobility tasks and 12,895 observations from persons who needed help with self-care tasks. Characteristics associated with new onset of mobility unmet needs were: age 80+, minority race, female sex, 2+ mobility limitations, 2+ chronic conditions, and dementia. Hospitalization during the yearly interval increased risk for new onset of unmet need for those with dementia (OR=3.01, 95%CI=2.62-3.44) and for those without dementia (OR=2.27, 95%CI=2.03-2.53). New onset of unmet need increased risk for nursing home or residential care placement (OR=1.63, 95%CI=1.31-2.03) and mortality (OR=2.10 95% CI =1.84-2.39) in the year following new onset of mobility unmet need. Results were similar for self-care daily activities. The findings inform the need for regularly assessing care needs, particularly after hospital discharge, with the goal of addressing new care needs to prevent new onset of unmet needs and its health consequences.

